# The Efficacy and Safety of Tirofiban Use in Endovascular Thrombectomy for Intravenous Thrombolysis Applicable Patients with Large Vessel Occlusion Stroke—a Post Hoc Analysis from the Direct-MT Trial

**DOI:** 10.1007/s00270-023-03540-9

**Published:** 2023-10-05

**Authors:** Yingying Zhang, Ping Zhang, Anyang Tao, Xinliang Wang, Jiangxian Ying, Zhimin Wang, Pengfei Yang, Yongwei Zhang, Lei Zhang, Zifu Li, Meng Zhang, Chenghua Xu, Jianmin Liu, on behalf of The Direct-MT Investigators

**Affiliations:** 1 Department of Neurology, Fudan University Zhongshan Hospital, Shanghai, China; 2 Department of Neurovascular Center, Naval Medical University Changhai Hospital, Shanghai, China; 3 Department of Neurology, Taizhou First People’s Hospital, 218 Hengjie Road, Taizhou, 318020 Zhejiang China; 4 Department of Neurosurgery, Liaocheng People’s Hospital of Shandong First Medical University, 67 West Dongchang Road, Liaocheng, 252200 Shandong China; 5 Department of Neurology, Naval Medical University Naval Medical Center of PLA, Shanghai, China

**Keywords:** Acute ischemic stroke, Large vessel occlusion, Intravenous thrombolysis, Endovascular thrombectomy, Tirofiban

## Abstract

**Purpose:**

The purpose of the study was to evaluate the efficacy and safety of tirofiban use in endovascular thrombectomy for intravenous thrombolysis applicable patients of large vessel occlusion stroke with data from Direct-MT trial.

**Materials and Methods:**

Direct-MT was the first randomized controlled trial to prove the non-inferiority of thrombectomy alone to bridging therapy (intravenous thrombolysis before thrombectomy) for large vessel occlusion stroke. Patients who underwent endovascular procedure were included and divided into thrombectomy-alone group and bridging therapy group. The effect of tirofiban use on 90 days MRS distribution, MRS 0–2 and mortality, successful reperfusion, the ASPECTS and outcome lesion volume of index stroke, re-occlusion of the treated vessel, futile recanalization and safety outcomes were further evaluated in both groups after adjustment for relevant confounding factors. The interaction between tirofiban and rt-PA was also assessed.

**Results:**

Of 639 patients included in this analysis, 180 patients underwent thrombectomy with tirofiban use (28.2%). Patients with tirofiban use had lower percentage of bridging therapy (41.1% vs 54.3%, *P* = 0.003), higher proportion of large artery atherosclerosis (*P* < 0.001) and more emergent stenting (30.56% vs 6.97%, *P* < 0.001). After adjustment for confounding factors, the 90-day modified Rankin Scale distribution, successful final recanalization rate, outcome lesion volume of index stroke on CT and intracranial hemorrhage risk showed no difference after tirofiban use in thrombectomy-alone group and in bridging therapy group. No interaction effect between tirofiban and rt-PA was detected.

**Conclusion:**

Based on data from Direct-MT trial, tirofiban is a safe medication for intravenous thrombolysis applicable patients with large vessel occlusion stroke undergoing thrombectomy.

**Level of Evidence:**

Level 3, cohort study of randomized trial.

## Introduction

Tirofiban, a non-peptide selective glycoprotein IIb/IIIa receptor inhibitor, can inhibit fibrinogen-dependent platelet aggregation and subsequent thrombosis [1]. It has been routinely applied in percutaneous coronary intervention for prevention of recurrent myocardial infarction and ischemic complications before its wide application in neurointervention [2, 3]. Tirofiban inhibits platelet aggregation in a dose-dependent manner and exerts the antiplatelet effect from 30 min after loading dose, with normalization of platelet function within no more than 4 h after treatment stop [4].

Tirofiban has been gradually accepted as an adjunctive therapy to thrombectomy for emergent large vessel occlusion (LVO) stroke, although its general efficacy and safety remained controversial [5–8]. In cases of intracranial atherosclerotic stenosis (ICAS)-related LVO stroke, tirofiban is effective and well-tolerated [9]. On the other hand, tirofiban, as an antiplatelet agent, can theoretically increase hemorrhagic risk after rt-PA infusion. However, tirofiban administration was reported for no increase in the occurrence of intracranial hemorrhage after intravenous thrombolysis (IVT) [10, 11]. The safety of tirofiban administration after IVT in stroke patients undergoing endovascular thrombectomy (EVT) is still indecisive. Meanwhile, with the progress of thrombectomy techniques, the successful recanalization rate has reached to over 80%, whether the tirofiban use might further improve the efficacy of thrombectomy is unclear.

Recently, the Endovascular Treatment With vs Without Tirofiban for Patients with Large Vessel Occlusion Stroke (RESCUE BT) trial revealed no difference in the 90 days modified Rankin Scale (MRS) distribution between two groups, but suggested a possible benefit of tirofiban use in the large artery atherosclerosis (LAA) subgroup [12]. The RESCUE BT trial enrolled patients undergoing EVT between 4.5 h and 24 h after symptoms onset, and patients with IVT were excluded. The question for the efficacy and safety of tirofiban use in IVT applicable patients was not answered by the RESCUE BT trial.

The Direct-MT trial was the first randomized controlled trial to prove the non-inferiority of thrombectomy alone to bridging therapy (intravenous thrombolysis before thrombectomy) for emergent LVO stroke of anterior circulation in endovascular treatment capable centers [13]. Patients within 4.5 h after stroke symptom onset were enrolled and assigned to thrombectomy-alone group or bridging therapy group randomly. In such a context, the effect of tirofiban use can be critically evaluated in thrombectomy-alone patients and in bridging therapy patients with comparable baseline data. This post hoc analysis aimed to provide reference to the efficacy and safety of tirofiban use in thrombectomy for emergent LVO stroke of IVT applicable patients.

## Material and Methods

### Patient Selection

The data of this study are available from the corresponding author upon reasonable request. Patient eligibility and the protocol of the Direct-MT trial (NCT03469206) have been reported previously [14]. There were 1586 patients screened for eligibility and 656 patients randomized. Of 656 patients, 327 patients allocated to the thrombectomy-alone group and 329 patients to the bridging therapy (intravenous thrombolysis before thrombectomy) group. After that, 11 patients in the thrombectomy-alone group and six in the bridging therapy group were excluded because of not undergoing catheter angiography. Finally, a total of 639 patients, with 316 in the thrombectomy-alone group and 323 in the bridging therapy group, were included in this post hoc analysis. This study was approved by all relevant local ethics committees and research boards. Written informed consent was obtained from all the patients or their legal representatives.

### Tirofiban Use

Tirofiban use was defined as periprocedural intravenous with or without intra-arterial use of the tirofiban if, (1) emergent stent angioplasty was unavoidable, and (2) vessel re-occlusion occurred or was expected after successful recanalization. The expected re-occlusion after successful recanalization indicated the antegrade flow in the treated vessel got deterioration after successful recanalization which was detected at any single run before the end of procedure. For tirofiban use, the loading dose was 0.1–0.4 ug/(kg min) for 30 min by intravenous infusion or combined with intra-arterial infusion, or 3-12 ug/kg intravenous bolus dose, then followed with intravenous maintenance dose of 0.1 ug/(kg min). Oral dual antiplatelets were prescribed for stenting patients after 24 h from IVT with the exclusion of intracranial hemorrhage by CT and overlapped with intravenous tirofiban for 6 h. For patients without stenting, single antiplatelet was prescribed in the same manner.

### Radiographic Variables Assessment

The location of occlusion was evaluated on baseline pre-procedure CTA and classified as ICA, M1 and M2 by the core laboratory. The extent of thrombus was quantified using the clot burden score [15]. The collateral grading system was scored on a 4-point scale of 0–3 and dichotomized into grades 0–1 and grades 2–3 [16].

### Primary and Secondary Outcomes

The primary outcome was 90 days MRS distribution shift. The secondary outcomes included good clinical outcome and mortality at 90 days, rates of successful reperfusion on final angiogram, the Alberta Stroke Program Early Computed Tomography score (ASPECTS) and outcome lesion volume of index stroke on follow-up CT, re-occlusion rate of the treated vessel on follow-up CTA and futile recanalization rate. The good clinical outcome was defined as MRS 0–2 at 90 days. Mortality was defined as MRS 6 at 90 days. Successful reperfusion was defined as final extended thrombolysis in cerebral ischemia (eTICI) 2b, 2c or 3 [17]. The outcome lesion volume was assessed on follow-up CT on days 5–7 using an automated algorithm [18]. Re-occlusion was defined as the total occlusion on follow-up CTA of the treated vessel (final eTICI > 0). Futile recanalization was defined as 90 days MRS > 2 despite a successful reperfusion (final eTICI ≥ 2b) [19–21].

### Safety Outcomes

Safety outcomes included rates of symptomatic intracranial hemorrhage (sICH), any ICH, large or malignant middle cerebral artery (MCA) infarction, infarctions in new territory at 5–7 days and femoral access complications. The sICH was defined according to the Heidelberg criteria [22].

### Statistical Analysis

Categorical variables were expressed as frequencies and percentages, continuous as mean (SD, standard deviation) for normal distribution or median (IQR, interquartile range) for non-normal distribution. Missing data were imputed with the use of multiple imputation by fully conditional specification regression for continuous variables or by fully conditional specification logistic regression for binary and ordinal variables. Categorical variables were tested using the Chi-square test. Continuous variables with normal distribution were tested using the t-test. Continuous variables with non-normal distribution were tested using the rank-sum test. Non-parametric testing of categorical variables was tested using Fisher’s exact test. Multivariable logistic regression analysis was applied to estimate the adjusted odds ratio (OR) for a shift in direction toward better outcome for with tirofiban vs without tirofiban in thrombectomy-alone group and bridging therapy group after adjustment for confounding factors. Confounding factors included age, sex, cause of stroke, the National Institutes of Health Stroke Scale (NIHSS) score at baseline, the time from stroke onset to randomization, the MRS score before stroke onset, cerebral collateral blood flow status, history of antiplatelet agents and emergent stenting. Adjusted OR was reported with 95% confidence interval (CI). The interaction between tirofiban and rt-PA was also evaluated after adjustment for confounding factors. Statistical testing was conducted at a two-tailed α level of 0.05. All the analyses were performed using the SAS software, version 9.2 (SAS Institute).

## Results

### Baseline and Clinical Data of Patients With/Without Tirofiban in Direct-MT Trial

**Fig. 1:**
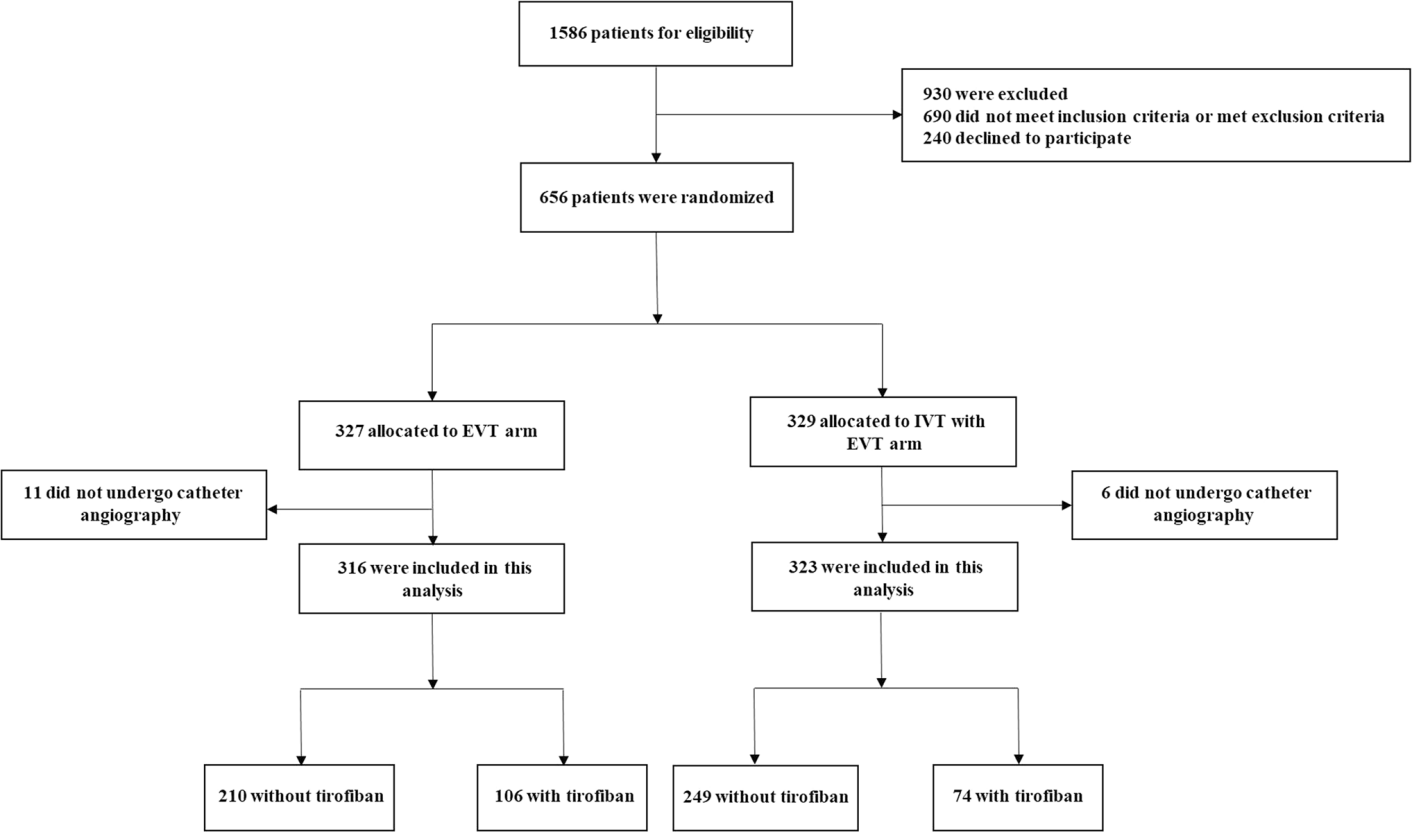
The flow diagram for patient selection

Overall, there were 639 patients included in this post hoc analysis. The flow diagram for patient selection is presented in Fig. 1. Among 639 patients, there were 180 (28.2%) patients with tirofiban use. Patients with tirofiban use were younger and more male sex. Lower percentage of bridging therapy (41.1% vs 54.3%, *P* = 0.003) and history of antiplatelet agents use (8.33% vs 16.1%, *P* = 0.011) were observed with tirofiban use. Difference in cause of stroke was detected, as more intracranial atherosclerosis and more ipsilateral extracranial ICA obstruction in patients with tirofiban use (*P* < 0.001). There was also more stenting in patients with tirofiban use (30.6% vs 6.97%, *P* < 0.001). Baseline data are presented in Table 1.

**Table 1:** Baseline and clinical data of patients with/without tirofiban in Direct-MT trial

Characteristic	Tirofiban−	Tirofiban +	*P* value
Overall	459	180	
Age, yr; median (IQR)	71 (62, 77)	67 (57.5, 74.5)	0.0009
Male sex	247 (53.81%)	114 (63.33%)	0.0290
Baseline NIHSS; median (IQR)	17 (13, 22)	16 (13, 21.5)	0.4166
Baseline ASPECTS; median (IQR)*	9 (7, 10)	9 (7, 10)	0.8589
Baseline SBP, mmHg; median (IQR)	144 (130, 160)	151 (132.5, 165.5)	0.0831
Baseline DBP, mmHg; median (IQR)	83 (75, 93)	85 (78, 96)	0.1196
With intravenous thrombolysis	249 (54.25%)	74 (41.11%)	0.0028
Onset to puncture time, min; median (IQR)	205 (160, 251)	201 (153.5, 240)	0.0907
Vascular risk factors			
Previous ischemic stroke	63 (13.73%)	24 (13.33%)	0.8965
Atrial fibrillation	227 (49.46%)	68 (37.78%)	0.0077
Diabetes mellitus	83 (18.08%)	38 (21.11%)	0.3795
Hypertension	274 (59.69%)	108 (60%)	0.9436
Hypercholesterolemia	18 (3.92%)	8 (4.44%)	0.7635
Smoking	94 (20.48%)	46 (25.56%)	0.1629
History of antiplatelet agents			
Aspirin	74 (16.12%)	15 (8.33%)	0.0105
Clopidogrel	23 (5.01%)	6 (3.33%)	0.3594
Ticagrelor	0 (0%)	0 (0%)	-
Cilostazol	0 (0%)	0 (0%)	-
Cause of stroke			< 0.0001
Cardioembolism	225 (49.02%)	59 (32.78%)	
Intracranial atherosclerosis	9 (1.96%)	36 (20%)	
Ipsilateral extracranial ICA obstruction	24 (5.23%)	39 (21.67%)	
Undetermined	201 (43.79%)	46 (25.56%)	
Location of occlusion^			0.3541
ICA	159 (35.10%)	65 (36.52%)	
M1	240 (52.98%)	95 (53.37%)	
M2	54 (11.92%)	17 (9.55%)	
Clot burden score; median (IQR)#	4 (2, 5)	4 (2, 5)	0.6354
Collateral status (0–1)	361 (78.65%)	136 (75.56%)	0.3975
Emergent stenting	32 (6.97%)	55 (30.56%)	< 0.001

Data are presented as number/total number (%), unless otherwise stated. *N* indicates number

*IQR* interquartile range, *NIHSS* National Institutes of Health Stroke Scale, *ASPECTS* Alberta Stroke Program Early Computed Tomography score, *SBP* systolic blood pressure and *DBP* diastolic blood pressure

*6 missing baseline ASPECTS (*n* = 6 in without tirofiban group)

^8 missing location of occlusion (*n* = 6 in without tirofiban group)

#8 missing clot burden score (*n* = 6 in without tirofiban group)

### Primary and Secondary Outcomes

After being adjusted for age, sex, cause of stroke, the NIHSS score at baseline, the time from stroke onset to randomization, the MRS score before stroke onset, cerebral collateral blood flow status, history of antiplatelet agents and emergent stenting, tirofiban use showed no benefit in primary outcome of 90 days MRS distribution shift and in secondary outcomes of 90 days MRS 0–2, 90 days mortality, successful reperfusion rate, follow-up ASPECTS, outcome lesion volume of index stroke, re-occlusion rate of the treated vessel and futile recanalization rate in thrombectomy-alone group and in bridging therapy group. No interaction was detected between tirofiban and rt-PA considering the primary and secondary outcomes.

### Safety Outcomes

Tirofiban use had no influence on the risk of sICH and any ICH in thrombectomy-alone group and in bridging therapy group. There was no interaction effect between tirofiban and rt-PA for ICH occurrence. No deterioration was detected in other safety outcomes. Relevant data are presented in Tables 2 and 3.

**Table 2:** Efficacy and safety of tirofiban use in patients underwent thrombectomy with or without intravenous thrombolysis

Outcome	Thrombectomy alone, adjusted OR* (95%, CI)	*P* value	Bridging therapy, adjusted OR* (95%, CI)	*P* value
*Primary outcome*				
90d MRS distribution	1.04(0.66,1.65)	0.87	1.15(0.68,1.96)	0.59
*Secondary outcome*				
90d MRS 0–2	1.28(0.68,2.39)	0.45	1.43(0.72,2.82)	0.31
90d MRS 6	1.72(0.87,3.41)	0.12	1.04(0.47,2.32)	0.92
Final eTICI ≥ 2b	1.01(0.54,1.87)	0.99	0.91(0.45,1.86)	0.80
Final eTICI ≥ 2c	1.17(0.69,1.98)	0.57	1.01(0.55,1.85)	0.98
Final eTICI 3	1.15(0.65,2.02)	0.63	0.87(0.45,1.67)	0.68
24-72 h ASPECTS	0.11(-0.56,0.79)	0.74	-0.20(-0.94,0.53)	0.59
5-7d ASPECTS	0.49(-0.18,1.17)	0.15	0.29(-0.47,1.05)	0.46
Outcome lesion volume	-18.05(-39.38,3.28)	0.10	-7.68(-30.22,14.86)	0.51
Re-occlusion rate on follow-up CTA	2.46(0.40,15.01)	0.33	0.21(0.01,3.69)	0.29
Futile recanalization rate	0.71(0.40,1.25)	0.23	0.52(0.27,1.01)	0.05
*Safety outcome*				
sICH	0.25(0.05,1.29)	0.10	0.14(0.02,1.23)	0.08
Any ICH	0.82(0.47,1.43)	0.48	0.88(0.47,1.64)	0.68
Large or malignant MCA infarction	1.27(0.59,2.69)	0.54	1.83(0.78,4.28)	0.16
Infarction in new territory at 5-7d	0.40(0.06,2.75)	0.35	1.96(0.33,11.60)	0.46
Femoral access complications	< 0.01(< 0.01, > 999.99)	0.94	< 0.01(< 0.01, > 999.99)	0.57

*MRS* modified Rankin Scale, *ASPECTS* Alberta Stroke Program Early Computed Tomography score, *eTICI* extended thrombolysis in cerebral ischemia and *sICH* symptomatic ICH

*Values were adjusted for age, sex, cause of stroke, the NIHSS score at baseline, the time from stroke onset to randomization, the modified Rankin Scale score before stroke onset, cerebral collateral blood flow status, history of antiplatelet agents and emergent stenting

**Table 3:** The interaction effect between tirofiban and rt-PA in Direct-MT trial

Outcome	Effect parameter	*P* value_interaction_
*Primary outcome*		
90d MRS distribution	Common odds ratio	0.86
*Secondary outcome*		
90d MRS 0–2	Odds ratio	0.95
90d MRS 6	Odds ratio	0.79
Final eTICI ≥ 2b	Odds ratio	0.36
Final eTICI ≥ 2c	Odds ratio	0.37
Final eTICI 3	Odds ratio	0.30
24-72 h ASPECTS	Beta	0.35
5-7d ASPECTS	Beta	0.40
Outcome lesion volume	Beta	0.34
Re-occlusion rate on follow-up CTA	Odds ratio	0.61
Futile recanalization rate	Odds ratio	0.40
*Safety outcome*		
sICH	Odds ratio	0.47
Any ICH	Odds ratio	0.95
Large or malignant MCA infarction	Odds ratio	0.31
Infarction in new territory at 5-7d	Odds ratio	0.30
Femoral access complications	Odds ratio	0.99

*MRS* modified Rankin Scale, *ASPECTS* Alberta Stroke Program Early Computed Tomography score, *eTICI* extended thrombolysis in cerebral ischemia and *sICH* symptomatic ICH

*Values were adjusted for age, sex, cause of stroke, the NIHSS score at baseline, the time from stroke onset to randomization, the modified Rankin Scale score before stroke onset, cerebral collateral blood flow status, history of antiplatelet agents and emergent stenting

## Discussion

Taking LVO stroke patients as a whole group, the efficacy and safety of tirofiban use in thrombectomy remained controversial. An observational study based on a single-center prospective registry study in China showed that tirofiban was not associated with higher sICH and leads to lower odds of deaths and better odds of long-term functional independence [23]. A multicenter registry from Korea revealed the safety of tirofiban use during endovascular therapy after IVT but failed to prove its efficacy [24]. In our study, tirofiban use showed no improvement in 90 days MRS distribution in LVO stroke patients undertaking thrombectomy with or without IVT. Also, it failed to bring benefit in successful recanalization rate enhancement and final infarct volume reduction. In Direct-MT trial, general successful recanalization rate reached at the level of 80%. If tirofiban brought potential contributions to the improvements in recanalization rate, it is really hard to be recognized with significance. Recently, a meta-analysis indicated that the use of tirofiban during IVT bridging thrombectomy might reduce the risk of re-occlusion of the treated vessel and 90 days mortality without increasing the risk of sICH and any ICH [25]. But no difference in re-occlusion rate after tirofiban use was detected in thrombectomy-alone group or in bridging therapy group according to our study. Given that we had adjusted confounding factors, such as the cause of stroke and emergent stenting, the additional benefit of tirofiban use in terms of preventing vessel re-occlusion other than in cases of LAA-related LVOs and of stent angioplasty was not claimed.

Several large-scale studies have investigated the efficacy and safety of tirofiban use in EVT for LVOs of LAA etiology. In a multicenter prospective study, 649 patients with LAA stroke were enrolled to evaluate the efficacy and safety of tirofiban use. Tirofiban was found to be associated with superior clinical outcomes in anterior circulation stroke and major stroke (NIHSS > 5) patients but with no cerebral hemorrhage increase [26]. In another study with 503 patients of LVOs retrospectively examined, intravenous tirofiban was associated with high recanalization rate and good outcome in both the whole cohort and the LAA subgroup [27]. Intravenous tirofiban infusion can also decrease the risk of early re-occlusion of the treated arteries with no increased risk of hemorrhage after angioplasty and stenting in ICAS-related LVOs [28].

As for the safety outcome, this post hoc analysis proved that tirofiban use increased ICH risk in neither group. No interaction effect existed between tirofiban and rt-PA. These results are consistent with the findings of a meta-analysis which included 722 patients with IVT bridging therapy from three trials, and 846 patients with IVT alone from seven studies published between 2001 and 2021. Pooled results showed that early tirofiban administration with IVT or bridging therapy did not increase the risks of sICH and ICH [29]. However, it was demonstrated that tirofiban, as an adjunct to thrombectomy, increased fatal bleeding risk in a dose-dependent manner [30]. In another nonrandomized study, tirofiban treatment increased risk of major ICH after endovascular thrombectomy, which might be the consequence of the sole intra-arterial administration of tirofiban [31]. Later, sole intra-arterial tirofiban was verified the association with higher hemorrhage rate and death rate as an adjunct to EVT [27]. In RESCUE BT trial, tirofiban was prescribed in a “routine” manner before EVT instead of in a flexible manner. Although no significant difference in the incidence of sICH between two groups (numerically higher for tirofiban group), the incidence of any ICH was increased in the tirofiban group [12]. In real-world practice, several noteworthy factors might influence the risk of cerebral hemorrhage after adjunctive tirofiban use during EVT. The first one is the actual time window of endovascular reperfusion. The hemorrhage risk increases with time period from symptom onset to reperfusion [32]. The second one is the severity of stroke, as the higher baseline NIHSS score can predict sICH risk after EVT independently [33]. The third one is the route and dosage of tirofiban use, which for now still lack consensus. In Direct-MT trial, the time period from symptom onset to reperfusion was strictly controlled, and tirofiban was administrated based on specific demand at a relatively low dosage level and mainly by transvenous route. Precise prescription of tirofiban in LVO stroke patients with acceptable hemorrhagic risk should be explored profoundly in the future.

Our study had several limitations. First, for now, neuro-interventionists still lack international guidelines for tirofiban use in the endovascular recanalization for LVOs. However, all of the operators from selected enrollment centers were of full experience in EVT, and the prescribed dosage of tirofiban in Direct-MT trial was within the range of the recommendations from 2019 Chinese expert consensus on clinical application of tirofiban in atherosclerotic cerebrovascular disease. Second, according to the design of Direct-MT trial, stent retriever was the primary device, and aspiration devices could be used as a secondary option if the initial reperfusion failed. This analysis, to some degree, represents the situation of tirofiban use in stent retrieval thrombectomy, in which the endothelial damages are regarded as unavoidable. Third, Direct-MT trial is a study based on Chinese population for whom the ICAS-related LVO is more common, but in western population, tirofiban is typically used in extracranial stenting evolved in tandem occlusion. The results of this analysis should not be generalized to all population. Fourth, given the nature of post hoc analysis, bias should be unavoidable. Well-designed prospective randomized controlled trials are needed to address this issue in the future. But we can still provide insight into the clinical practice and future research of tirofiban use in thrombectomy for IVT applicable patients with emergent LVO stroke.

## Conclusion

Based on data from Direct-MT trial, tirofiban is a safe medication for IVT applicable patients with LVO stroke undergoing thrombectomy. However, no matter with or without IVT, tirofiban use showed no benefits in achieving favorable outcomes.

## Abbreviations

**LVO**: Large vessel occlusion
**ICAS**: Intracranial atherosclerotic stenosis
**IVT**: Intravenous thrombolysis
**EVT**: Endovascular thrombectomy
**NIHSS**: National Institutes of Health Stroke Scale
**MRS**: Modified Rankin Scale
**ASPECTS**: Alberta Stroke Program Early Computed Tomography score
**eTICI**: Extended thrombolysis in cerebral ischemia
**sICH**: Symptomatic intracranial hemorrhage
**LAA**: Large artery atherosclerosis
